# Oral Health in Patients Hospitalized Because of Ischemic Stroke

**DOI:** 10.3390/jcm13154556

**Published:** 2024-08-04

**Authors:** Anna Dziewulska, Wioletta Pawlukowska, Alicja Zawiślak, Marta Masztalewicz, Katarzyna Grocholewicz

**Affiliations:** 1 Department of Interdisciplinary Dentistry, Pomeranian Medical University, 70-111 Szczecin, Poland; anna.dziewulska@pum.edu.pl (A.D.); alicja.zawislak@pum.edu.pl (A.Z.); 2 Department of Neurology, Pomeranian Medical University, 71-252 Szczecin, Poland; wioletta.pawlukowska@pum.edu.pl (W.P.); marta.masztalewicz@pum.edu.pl (M.M.); 3 Department of Maxillofacial Orthopedics and Orthodontics, Institute of Mother and Child, 01-211 Warsaw, Poland

**Keywords:** stroke, oral health, dentition, periodontium

## Abstract

**Background/Objectives**: Maintenance of good oral health is relevant to overall health and quality of life. Results of many analyses showed that stroke patients had worse oral health than the control population. The aim of this study was a clinical assessment of oral condition in post-stroke patients and a healthy population. **Methods**: Oral health was assessed in stroke patients on the first day of ischemic stroke, and in a control group of healthy subjects. The number of teeth, the presence of active carious foci, fillings, and prosthetic restorations were evaluated. To assess oral hygiene, the Approximal Plaque Index (API) was used. In periodontal examinations, the presence of dental deposits, the depth of the existing periodontal pockets, tooth mobility, and the Sulcus Bleeding Index (SBI) during probing were assessed. **Results**: Significantly higher mean values of Decayed Teeth (DT), Missing Teeth (MT), and Decayed, Missing, and Filled Teeth (DMFT) indices were recorded in the study group. The incidence of dental caries, API, and SBI was also significantly higher in the study group. The study and control groups did not differ significantly in the average number of pockets 3 mm deep and deeper and in the frequency of having prosthetic restorations. **Conclusions**: Oral health and the level of oral hygiene in patients hospitalized because of ischemic stroke, in comparison with that in a healthy population, is not satisfactory. Active interdisciplinary collaboration between various medical specialists in the therapy of patients with general illnesses, including stroke, is strongly recommended.

## 1. Introduction

Oral health, including periodontal status, is an important factor in maintaining good overall health and high quality of life. Negligence in oral hygiene in healthy persons may lead to dental caries and gum diseases [1]. Poor oral status can predispose hospitalized patients to developing serious problems such as pneumonia and other infections, as well as reduced nutrient intake, which in turn may increase morbidity and longer stay in the hospital ward [2,3,4,5,6,7]. One of the most prevalent diseases in old age is stroke. In stroke survivors, oral and swallowing functions usually decrease, which may also be due to the deterioration of brain function [8]. Persons who have experienced a stroke may suffer from the following problems: difficulties in moving and communicating, neurological deficits, pain, and depression [9,10]. Stroke, to a large extent, affects a person’s ability to care for themselves and increases their dependence on others to perform daily routine and oral hygiene activities. It is estimated that up to 75% of stroke survivors may be unable to brush their teeth as a result of disabilities in cognitive and physical health [11]. Motor dysfunction, which typically occurs in stroke, may lead to dysphagia, and coupled with poor oral hygiene, significantly enhances the risk of aspiration pneumonia. Acute stroke complications such as aspiration pneumonia or respiratory-related problems have a detrimental effect on subsequent prognosis [12]. Causes of aspiration pneumonia are associated with the following problems: saliva aspiration with bad oral hygiene, aspiration of food remains due to abnormal swallowing function and coughing, and reduced immunity. Dysphagia is almost inevitable, both in the acute phase of stroke and in the case of patients fed by tube. In this group of patients, subclinical aspiration of saliva, which is contaminated with microorganisms, occurs during sleep, so proper oral hygiene from the early stage is essential [13,14]. Many researchers have noticed a relationship between bad mouth condition and infections and long-term general diseases, including ischemic stroke [15,16,17]. It is commonly known that poor oral status can impact overall health, whereas systemic diseases can manifest themselves in the oral cavity or oral mucosa [18,19].

Comparisons of oral health in stroke patients with that in healthy control groups has been the subject of many recent metanalyses. The results revealed that stroke patients had worse oral and periodontal health, and less frequent dental appointments, in comparison with the control group [20,21,22]. Further research on post-stroke oral health problems and the implementation of effective management strategies are needed. More future research should analyze the potential association between oral health and neglected oral hygiene [23]. Thus, to support medical professionals, there is a need for more research on this topic. Accordingly, the present study attempted to estimate and compare the conditions of oral health in post-stroke patients during hospital stays with that in healthy individuals.

## 2. Materials and Methods

The study was designed as a prospective, open-label, nonrandomized clinical trial in a single center, approved by the Bioethics Committee at the Pomeranian Medical University in Szczecin (decision reference No. KB-0012/06/10) and performed in accordance with the Declaration of Helsinki. The study was registered at ClinicalTrials.gov, with identifier NCT06332846 and a registration date of 26 March 2024, and all methods were carried out in accordance with the appropriate guidelines and regulations. The study group consisted of 59 patients with rimary ischemic stroke, with symptoms from the anterior cerebral artery (basin of the internal carotid artery), and with severe neurological defects (minimum 3 points according to National Institute of Health Stroke Scale (NIHSS)) [24].

The exclusion criteria were as follows:

- Disturbances in consciousness, aphasia, or mental disorders, preventing the provision of informed consent;
- Surgery of the salivary glands, disrupting the secretion of saliva;
- Diseases that disrupt salivary secretion (diabetes mellitus, Sjogren’s syndrome, or conditions developed after radiotherapy in the area of the salivary glands).

The control group patients (59 individuals), matched for age and gender, were selected among patients of the Department of Interdisciplinary Dentistry at Pomeranian Medical University in Szczecin. The participation of all individuals in this research was voluntary. All patients provided written informed consent.

Medical history, physical examinations with an evaluation of neurological status, and computed tomography (CT) scans of the head were used to make a diagnosis. The intensity of stroke-related neurological manifestations was assessed on the first day of ischemic stroke (admission) using the NIHSS scale [24]. 

Oral health was assessed on the first day of ischemic stroke in each patient included in the study. The examination was conducted with the use of a dental mirror and a calibrated periodontal probe, in daylight (study group) or under artificial lightning (control group), following the World Health Organization criteria [25]. The teeth examined were moist and had not been professionally cleaned beforehand. Each available surface of all teeth was evaluated. 

The evaluation included the following elements: the number of missing teeth, active caries lesions, and existing restorations. Each tooth was assessed and classified as follows: decayed (DT), extracted because of a carious process (MT), or filled due to caries (FT). The data collected from the dental examination were used to calculate the DMFT index (DT + MT + FT), and expressed the occurrence of dental caries. Active carious lesions were recorded when visible tissue loss, undermined enamel, or a detectably softened area was present. A probe was used to verify visual evidence of caries. Teeth extracted due to caries complications were considered missing (MT), which was verified during the review of medical history. When there was at least one permanent restoration placed to treat a carious process, the tooth was qualified as filled (FT). The simultaneous presence of filling and carious lesions in the tooth resulted in a determination of the tooth as DT. 

The presence of prosthetic restorations was also assessed (crowns, bridges, removable/metal framework prostheses).

The Approximal Plaque Index (API) was used to assess oral hygiene [26]. The presence of plaque was determined in interproximal areas (at least 10–12 areas). The API was determined as a percentage, according to the proportion of the number of interproximal areas with plaque to the number of all assessed interproximal areas. API values between 100 and 70% indicate improper oral hygiene, values between 70 and 40% indicate average oral hygiene, values between 39 and 25% indicate quite-good hygiene, and values below 25% indicate optimal oral hygiene.

The periodontal examination included assessments of the following: the presence of dental calculus (present or absent), the depth of the existing periodontal pockets (each tooth was evaluated with four surfaces: bucally, lingually, mesially and distally), tooth mobility according to Hall [27] (0—no mobility; 1—labio-lingual mobility up to 1 mm; 2—labio-lingual mobility up to 2 mm; 3—labio-lingual and vertical mobility) with the use of Periotest (Medizintechnik Gulden, Modautal, Germany), and the Sulcus Bleeding Index during probing (SBI) [28]. The SBI is an inflammatory gingival index in which bleeding is measured from four gingival units (mesial and distal papillary units and labial and lingual marginal units) using a periodontal probe with a 0.5 mm diameter tip. The scoring range around eight anterior teeth (four maxillary and four mandibular) is from 0 (healthy appearance and no bleeding on probing) to 5 (spontaneous bleeding with marked swelling and a change in color). The API and SBI are used in dental practice to register patients’ plaque control and gingival inflammation. Both indices focus on interproximal areas that are, in general, plaque-retentive and susceptible to inflammation.

For the evaluation of quantitative variables, an arithmetic mean was used, taking into account the minimum and maximum values and the standard deviation. Regarding the analysis of qualitative variables, the percentage values and the number of the variables were used. Due to the distribution of the analyzed data deviating from the normal distribution (Shapiro–Wilk test, *p* < 0.05), groups of variables were compared using nonparametric tests, including the Mann–Whitney U-test for two groups. The Wilcoxon pair order test was used to compare the dependent variables, and Pearson’s chi-square test for 2 × 2 tables was used to analyze the qualitative variables. The significance level for the analyzed variables was *p* = 0.05. For statistical analysis, we used statistical language R (version 4.1.1; [29]) in the Windows 10 Pro 64 bit (compilation 19045) system, by using the packages report (version 0.5.1.3; [30]), gtsummary (version 1.6.2; [31]), readxl (version 1.3.1; [32]) and dplyr (version 1.0.10; [33]).

## 3. Results

The results were analyzed for N = 118 patients, 62 female (52.5%) and 56 male (47.5%), aged Mdn = 69 years (Min = 45 years, Max = 89 years). The trial consisted of a study group and a control group; *n*_study_ = *n*_control_ = 59. The study included sociodemographic data and results of clinical parameters.

The characteristics of the sample regarding the sociodemographic data by group are shown in Table 1. The data in Table 1 show that the ages in the control group were significantly higher compared to those in the study group (*p* = 0.027); in addition, the percentage of women in the control group was significantly higher than that in the study group (the percentage of men in the control group was significantly lower; *p* = 0.027).

Table 2 presents the results of clinical parameters in the study and control groups. The data showed the absence of edentulism in both groups.

Significantly higher mean values of DT, MT, and DMFT indices were recorded in the study group (*p* = 0.007, and *p* < 0.001); in turn, the mean values of the FT index were significantly higher in the control group (*p* < 0.001). 

The groups did not differ significantly in the frequency of having prosthetic restorations. However, in the control group, there was a significantly higher percentage of fixed dentures, at *p* = 0.001, specifically crowns, at *p* = 0.004, and bridges, at *p* = 0.018. Regarding removable dentures (both partial and metal framework dentures), no significant differences were found between the groups.

Calculus was present significantly more often in stroke patients (*p* < 0.001), the API and SBI in the study group were significantly higher compared to those in the control group, and gingivitis occurred significantly more often in the study group (*p* = 0.001).

The study and control groups did not differ significantly in terms of the average number of pockets with a depth of 3 mm and deeper; however, the number of pockets less than 3 mm deep was significantly higher in the control group (*p* = 0.001).

During the examination of tooth mobility, the number of teeth examined in the control group was significantly higher than that in the study group (*p* = 0.001). The mean distribution of I-degree mobility was the same in both groups, however, the numbers of mobility parameters’ II and III degrees were significantly higher in the study group (*p* = 0.001). 

## 4. Discussion

The study revealed that the patients hospitalized due to ischemic stroke had worse oral status and hygiene than the patients from the control group. 

Stroke patients had more missing and decayed teeth; however, they had less filled teeth than healthy patients. With fewer teeth present, stroke patients are more likely to have calculus, bleeding gums, and gingivitis due to poorer oral hygiene. Tooth mobility of the II and III degrees is also more frequent in stroke patients. Patients in the control group were more likely to have fixed prostheses than stroke patients. These results suggest a higher level of oral care in the control group before a stroke occured. Sinha et al. [34] also observed a high incidence of periodontitis (100%) and caries (79%) in stroke patients. Takagi et al. [35] studied associations between oral health conditions and short-term functional outcomes in hospitalized patients with acute ischemic stroke. They observed that elderly people admitted to the hospital for acute ischemic stroke had poor oral health status, which was associated with worse short-term functional outcomes, such as increased disability, dysphagia, and longer hospital stays. The study by Wu et al. [36] confirmed the association of stroke severity with the level of oral health. They found that in stroke patients, 55% had a changed or unhealthy condition of gums and soft tissue, and only 8.3% had healthy teeth. 

It is estimated that 20–85% of the population suffers from periodontal problems [37,38]. In the analyzed groups, gingivitis was found significantly more often in stroke patients than in the healthy subjects. Pawlukowska et al. [39], examining stroke patients with and without periodontitis, diagnosed periodontal diseases in 36% of all patients. They observed that neurological status was severely worse in patients suffering from periodontal diseases than in other patients. Tooth loss as a symptom of chronic periodontitis indicates poor oral health and is pathogenetically linked to ischemic stroke risk [40,41,42]. Many studies indicate that both periodontal problems and poor oral health increase the likelihood of ischemic stroke [42,43,44,45]. The impact of periodontal diseases on stroke severity and subsequent disability has been the subject of some analyses [40,45,46].

Słowik et al. [40] observed that people with periodontal diseases or edentulism had worse neurological conditions at the beginning in comparison with patients without periodontal problems. According to Leira et al. [45], periodontitis contributes to disability after a stroke episode. Furthermore, poor oral health in stroke patients may be related to an increase in the risk of respiratory infections [47,48,49]. In the study by Pawlukowska et al. [39], a higher incidence of infections was more often observed in patients with periodontal problems. In the literature, more publications exist that indicate an association of poor oral health in stroke patients with respiratory infections [23,47]. It has been also reported that the occurrence of pneumonia in stroke patients is more common in those with larger caries lesions and worse oral hygiene [23]. Undoubtedly, maintaining good oral health, for patients with acute stroke, seems to be an important part of preventing respiratory infections in these patients.

Subjects with acute stroke are susceptible to phlegm and its retention, xerostomia (dry mouth), and improper oral hygiene because of deteriorated oral functions and post-stroke disability [50]. Xerostomia may result in the following complications: discomfort, pain when eating, infections of the oral mucosa, caries, and loose teeth [51]. Early and appropriately selected dental treatment can significantly improve oral health [13]. It has been also suggested that close cooperation with dentists and dental hygienists can help to prevent various acute stroke complications, such as aspiration pneumonia [52]. The use of prostheses by older patients makes their oral environment more complex. Enhanced oral hygiene procedures are usually recommended [53]. It has been reported that oral care using chlorhexidine agents can limit ventilated aspiration pneumonia by about 10–30% in patients undergoing cardiac surgery [54,55,56]. Maintaining high standards of oral hygiene is certainly an essential intervention for preventing aspiration pneumonia and xerostomia.

While most patients are aware of the importance of good oral health, they are unaware that poor oral health can affect their overall health. In a study by Ajvani et al. [57], many subjects had active dental and gum disease conditions. This complex problem requires dentists’ participation within a multidisciplinary team and that they co-work with patients and their families because stroke patients are at risk of a deterioration in their oral health [20,35,36,57]. Patients with disabilities can face difficulty accessing dental treatment. In addition to the limited availability of dental services, there are some additional hindering factors: physical limitations or psychological, sociological, or economic factors [58]. 

According to the study by Ajwani’s et al. [57], stroke patients experience various oral problems and show low awareness of how the latter affect their general health. The authors concluded that, because there are currently no oral healthcare programs aimed at post-stroke patients, it would be appropriate to design such programs and introduce them into the hospital care of these patients. 

Stroke survivors have worse oral health, as reflected by many parameters, for example, tooth loss, tooth decay, and periodontal status, so oral health and hygiene can be defined as key factors in this group of patients [15,16,20,59]. In an observation by Gerreths et al. [60], oral hygiene and the condition of gums were not satisfactory at the beginning, but improved slightly by the end of the stay in the rehabilitation unit, and improved as overall health improved. In our study, oral hygiene in stroke patients was significantly worse than that in the control group. Wu et al. [36] also observed unsatisfactory oral cleanliness in 92.2% of stroke patients. Undoubtedly, oral care is essential in the prevention of dental caries and other oral disorders, which can be a source of bacterial invasion [61], and particular attention to oral care is needed in this group of patients.

The strength of this study lies in its comprehensive assessment of oral health (including both teeth and periodontal status) and oral hygiene. Only patients who had experienced ischemic stroke were considered in our work; therefore, the study group was homogenous. Medical examination was performed by a multi-specialist medical team including a dentist (for oral assessment) and neurologist (for physical assessment). However, there are several limitations to the study. Our sample size was relatively small and we encountered some difficulties with dental examinations and communication with post-stroke patients who may have lied about their oral hygiene, which may have affected the data collected Periodontal examination was performed in both groups only one time, and because of the lack of information about previous periodontal conditions and the serious issue of patients lying about their oral hygiene, an evaluation of clinical attachment and the % of bone loss was impossible. Because of these difficulties, we could not use the latest periodontal classification and had to interview patients regarding medications taken and symptoms of xerostomia. We did not take radiographs of patients’ teeth for the diagnosis of interproximal caries, which may have caused some carious lesions to be underestimated.

## 5. Conclusions

The oral health and level of oral hygiene in patients hospitalized because of ischemic stroke are not satisfactory, and worse than those in the control group. During routine dental examinations, dentists should instruct every patient on oral hygiene, including hygiene practices for dentures. Moreover, the data in the literature reveal insufficient hospital staff training in oral hygiene methods and oral assessment. Instructions on oral care and prevention should therefore also be given to nurses, nursing students, and health care assistants by qualified dentists. In summary, active interdisciplinary collaboration between various medical specialists in the therapy of patients with general illnesses, including stroke, is strongly recommended. 

## Figures and Tables

**Table 1:** Characteristics of sociodemographic data of study and control groups.

Parameter	N	Group		*p*
		Study *n* = 59	Control *n* = 59	
Age [years]	118	66 (59.5, 72) ^2^	70 (64.5, 73.5) ^2^	0.027 ^3^
Sex	118			0.027 ^4^
Female		25 (42.4%) ^1^	37 (62.7%) ^1^	
Male		34 (57.6%) ^1^	22 (37.3%) ^1^	

^1^ *n* (%); ^2^ Mdn (median), (Q1, Q3—first quartile, third quartile); ^3^ Wilcoxon rank sum test; ^4^ Pearson’s chi-square test.

**Table 2:** Distribution of clinical parameters by group with estimation of intergroup differences.

Parameter	N	Group		*p*
		Study *n* = 59	Control *n* = 59	
Edentulism	118	0 (0, 0) ^2^	0 (0, 0) ^2^	*n*/d
DT	118	2 (1, 5.5) ^2^	1 (0, 2) ^2^	0.007 ^3^
MT	118	21 (9.5, 27) ^2^	9 (5.5, 15.5) ^2^	<0.001 ^3^
FT	118	2 (0, 6) ^2^	8 (5, 12) ^2^	<0.001 ^3^
DMFT	118	28 (22, 31) ^2^	22 (17.5, 27) ^2^	<0.001 ^3^
Prosthetic restoration	118			0.062 ^4^
no		30 (50.8%) ^1^	20 (33.9%) ^1^	
yes		29 (49.2%) ^1^	39 (66.1%) ^1^	
Fixed	118			<0.001 ^4^
no		50 (84.7%) ^1^	30 (50.8%) ^1^	
yes		9 (15.3%) ^1^	29 (49.2%) ^1^	
Removable	118			0.573 ^4^
no		34 (57.6%) ^1^	37 (62.7%) ^1^	
yes		25 (42.4%) ^1^	22 (37.3%) ^1^	
Crowns	118			0.004 ^4^
no		50 (84.7%) ^1^	36 (61%) ^1^	
yes		9 (15.3%) ^1^	23 (39%) ^1^	
Bridges	118			0.018 ^4^
no		55 (93.2%) ^1^	46 (78%) ^1^	
yes		4 (6.8%) ^1^	13 (22%) ^1^	
Partial dentures	118			0.296 ^4^
no		41 (69.5%) ^1^	46 (78.0%) ^1^	
yes		18 (30.5%) ^1^	13 (22%) ^1^	
Metal framework partial dentures	118			0.282 ^4^
no		53 (89.8%) ^1^	49 (83.1%) ^1^	
yes		6 (10.2%) ^1^	10 (16.9%) ^1^	
API (%)	118	100 (80, 100) ^2^	60 (35, 80) ^2^	<0.001 ^3^
Calculus	118			<0.001 ^4^
no		8 (13.6%) ^1^	28 (47.5%) ^1^	
yes		51 (86.4%) ^1^	31 (52.5%) ^1^	
SBI [%]	118	100 (100, 100) ^2^	0 (0, 20) ^2^	<0.001 ^3^
No data		1	0	
Gingivitis	118			<0.001 ^5^
no		1 (1.7%) ^1^	32 (54.2%) ^1^	
yes		57 (98.3%) ^1^	27 (45.8%) ^1^	
No data		1	0	
Pockets up to 3 mm	118	0 (0, 1) ^2^	3 (0.5, 8) ^2^	<0.001 ^5^
No data		1	0	
Pockets 3–6 mm	118	0 (0, 1) ^2^	0 (0, 2) ^2^	0.199 ^3^
No data		1	0	
Pockets above 6 mm	118	0 (0, 0) ^2^	0 (0, 0) ^2^	0.208 ^3^
No data		1	0	
Number of examined teeth(mobility)	118	10 (4.5, 18) ^2^	21 (14.5, 24) ^2^	<0.001 ^3^
Tooth mobility—grade 0	118	4 (1.5, 11.5) ^2^	19 (7.2, 24) ^2^	<0.001 ^3^
No data		0	1	
Tooth mobility—grade I	118	2 (0.5, 4) ^2^	0 (0, 4) ^2^	0.175 ^3^
Tooth mobility—grade II	118	1 (0, 1.5) ^2^	0 (0, 0) ^2^	<0.001 ^3^
Tooth mobility—grade III	118	1 (0, 1) ^2^	0 (0, 0) ^2^	<0.001 ^3^

^1^ *n* (%); ^2^ Mdn (median), (Q1, Q3—first quartile, third quartile); ^3^ Wilcoxon rank sum test; ^4^ Pearson’s chi-square test; ^5^ Fisher’s exact test.

## Data Availability

All data analyzed in this study are available from the corresponding author on reasonable request.
